# Host factors against plant viruses

**DOI:** 10.1111/mpp.12851

**Published:** 2019-07-08

**Authors:** Hernan Garcia‐Ruiz

**Affiliations:** ^1^ Nebraska Center for Virology, Department of Plant Pathology University of Nebraska‐Lincoln Lincoln NE 68503 USA

**Keywords:** antiviral defence, host factors, virus–host interactions, virus resistance

## Abstract

Plant virus genome replication and movement is dependent on host resources and factors. However, plants respond to virus infection through several mechanisms, such as autophagy, ubiquitination, mRNA decay and gene silencing, that target viral components. Viral factors work in synchrony with pro‐viral host factors during the infection cycle and are targeted by antiviral responses. Accordingly, establishment of virus infection is genetically determined by the availability of the pro‐viral factors necessary for genome replication and movement, and by the balance between plant defence and viral suppression of defence responses. Sequential requirement of pro‐viral factors and the antagonistic activity of antiviral factors suggest a two‐step model to explain plant–virus interactions. At each step of the infection process, host factors with antiviral activity have been identified. Here we review our current understanding of host factors with antiviral activity against plant viruses.

## Introduction

**Figure 1:**
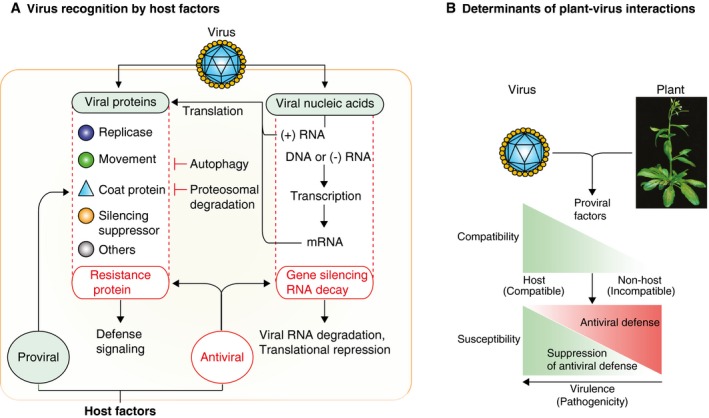
Genetic determinants of plant–virus interactions. (A) Viruses encode proteins to execute all parts of the infection cycle. Their expression is dependent on the host RNA translation machinery. Their activity requires host factors (pro‐viral) and resources. Antiviral immunity consists of host factors that target viral proteins or nucleic acids to restrict virus infection. (B) A two‐step model in plant–virus interactions. Compatibility is determined by the availability of pro‐viral host factors. Susceptibility is determined by the balance between antiviral defence and suppression of antiviral defence.

Infection of a plant by a virus initiates in a single cell. Viral proteins are synthesized by the host cell before genome replication, virion formation and movement to a neighbouring cell. The cycle is repeated at every newly infected cell (Nelson and Citovsky, 2005). Using the vascular system, plant viruses move long distances to infect tissues away from the initial site of infection, such as roots and young leaves (Heinlein, 2015; Wan *et al.*, 2015). Multiple genetic analyses have shown that the entire infection cycle, including virus replication and movement, is genetically determined by viral and cellular factors that synchronize their activities in time and space (Fig. 1A) (Diaz *et al.*, 2015; Hofius *et al.*, 2007; Laliberte and Zheng, 2014; Li *et al.*, 2016; Sasvari *et al.*, 2018; Zhang *et al.*, 2018).

Plant–virus combinations could result in an incompatible or compatible interaction. Incompatible interactions occur between a virus and a non‐host plant, are characterized by the absence of virus infection and may be explained by the lack of cellular factors essential for the virus to replicate or move, antiviral defence or a combination (Fig. 1B) (Jaubert *et al.*, 2011; Lellis *et al.*, 2002). In contrast, compatible interactions occur between a virus and a susceptible host, are characterized by the establishment of virus infection and indicate the presence of pro‐viral cellular factors and resources necessary for virus infection and movement. Infection may spread through the entire plant, parts of the plant or be limited to the vascular system or the initially infected organ (Calvo *et al.*, 2014a, 2014b; Lv *et al.*, 2017; Otulak‐Koziel *et al*., 2018).

In susceptible hosts the absence of critical pro‐viral host factors results in the absence of infection and reduced virus replication, movement or both (Hofius *et al.*, 2007; Lellis *et al.*, 2002; Wang and Nagy, 2008). Accordingly, the absence of pro‐viral factors may turn a susceptible host into a non‐host, as is the case with resistant cultivars, landraces or ecotypes within a susceptible species (Hashimoto *et al.*, 2016; Lellis *et al.*, 2002). Because their presence conditions susceptibility, while their absence results in immunity or resistance, several terms have been used to describe host genes with pro‐viral activity, such as loss of susceptibility, recessive resistance or susceptibility genes (Garcia‐Ruiz, 2018; Hashimoto *et al.*, 2016).

Interestingly, susceptible hosts harbour factors with antiviral activity (Diaz‐Pendon *et al.*, 2007; Kushner *et al.*, 2003; Panavas *et al.*, 2005; Scholthof *et al.*, 2011). To establish infection, viruses escape from or suppress antiviral defence activated by viral proteins or nucleic acids, particularly RNA (Fig. 1A) (Garcia and Pallas, 2015; Gorovits *et al.*, 2016). With or without a hypersensitive reaction, the defence response restricts essential parts of the infection cycle, such as viral RNA translation, virus replication or movement, resulting in reduced virus accumulation and/or a delay in the establishment of systemic infection. Symptoms may or may not develop (Donze *et al.*, 2014; Garcia‐Ruiz *et al.*, 2018; Korner *et al.*, 2018).

Multiple genetic analyses have shown that the outcome of plant–virus interactions is genetically determined by viral factors, host factors and their interaction (Fig. 1A) (Chisholm *et al.*, 2001; Jaubert *et al.*, 2011; Kushner *et al.*, 2003; Lellis *et al.*, 2002; Panavas *et al.*, 2005). Consistent with this model, for all parts of the infection cycle, at least one host gene with antiviral activity has been identified (Table 1). Here I review our current understanding of host factors with antiviral activity against plant viruses. Their antagonistic activity is presented following sequential parts of the infection cycle.

**Table 1:** Representative host factors with antiviral activity against plant viruses.

Host factor	Cellular function	Virus	Viral factor	Host	Technique	Reference
Viral RNA translation						
APUM5	mRNA binding	CMV, TuMV	mRNA	*Arabidopsis thaliana*	T‐DNA mutant screen	Huh *et al.* (2013)
NIK1	Receptor‐like kinase	CaLCuV	NSP	*A. thaliana*	Genetic analysis	Zorzatto *et al.* (2015)
Virus replication complex formation						
PAH1	Phospholipid biosynthesis	BMV, TBSB	1a, p33	Yeast and *Nicotiana benthamiana*	Genetic analysis	Chuang *et al.* (2014); Zhang *et al.* (2018)
Accumulation or activity of the replication proteins						
Beclin1 (ATG6)	Autophagy	TuMV	NIb	*N. benthamiana A. thaliana*	Autophagosome marker, yeast two‐hybrid	Li *et al.* (2018)
Tm‐1	NA	ToMV	130K	*Solanum lycopersicum*	Cell fractionation and mass spectrometry	Ishibashi *et al.* (2007)
TARF	Ubiquitination	TMV	126K	*Nicotiana tabacum*	Yeast two‐hybrid, VIGS	Yamaji *et al.* (2010)
Ubiquiting‐proteosome system	Protein degradation	TYMV	RdRp	*A. thaliana*	Pulse‐chase labelling	Camborde *et al.* (2010)
Rsp5p	Ubiquitination	TBSV	P92	Yeast	Proteomics	Barajas *et al.* (2009)
PVR4	NA	PepMV, PVY	NIb	*Capsicum annum*	Transient expression	Kim *et al.* (2015)
mRNA stability						
DCP1, DCP2, XRN4, PARN	mRNA decay	TuMV	mRNA	*N. benthamiana*, *A. thaliana*	Genetic analysis	Li and Wang (2018)
XRN4	mRNA decay	TBSV	mRNA	Yeast and *N. benthamiana*	Genetic mutation, VIGS	Jaag and Nagy (2009)
XRN4	mRNA decay	TMV	mRNA	*N. benthamiana*	VIGS	Peng *et al.* (2011)
DCP1	mRNA decay	TRV	mRNA	*A. thaliana*	Genetic mutation	Ma *et al.* (2015)
Virus movement						
ESC1 (AtPiezo)	Mechanosensitive ion channel	CMV, TuMV	NA	*A. thaliana*	EMS mutagenesis	Zhang *et al.* (2019)
RTM1, RTM2, RTM3	Protein binding	TEV	CP	*A. thaliana*	GUS or GFP‐fusion constructs	Chisholm *et al.* (2001); Decroocq *et al.* (2009)
KELP	Transcription coactivator	ToMV	p30	*N. benthamiana*	Transient expression	Sasaki *et al.* (2009)
BTR1	mRNA binding	ToMV	Genomic RNA	*A. thaliana*	Immunoprecipitation and mass spectrometry	Fujisaki and Ishikawa (2008)
Rsv3	NA	SMV	CI	*Glycine max*	Genetic analysis	Zhang *et al.* (2009)
Rsv4	NA	SMV	NA	*G. max*	Genetic analysis	Ma *et al.* (1995)
Ny‐1	NA	PVY	NA	*Solanum tuberosum*	Hybrids between resistant and susceptible cultivars	Lukan *et al.* (2018); Szajko *et al.* (2008)
Antiviral gene silencing						
DCL, AGO, RDR, SGS, DRB	gene silencing	CaMV, CymRSV, MNSV, PMMoV, ORMV, TuMV, SCMV, MCMV, CMV, PVA, TCV, TBSV, TSWV, PVX, ToRSV, RSV, TRV, TYLCV, WMV	RNA	*A. thaliana*, *N. benthamiana Zea mays*, *Oryza sativa Cucumis melo*, *S. lycopersicum*	Genetic analysis	Blevins *et al.*, (2006); Brosseau and Moffett (2015); Diaz‐Pendon *et al.* (2007); Donaire *et al.* (2009); Garcia‐Ruiz *et al.* (2010, 2015); Jaubert *et al.* (2011); Karran and Sanfacon (2014); Ludman *et al.* (2017); Qu *et al.* (2008); Raja *et al.* (2014); Scholthof *et al.* (2011); Wu *et al.* (2015); Xia *et al.* (2016))
Ty‐1	RNA‐dependent RNA polymerase	ToYLCV	Genomic DNA	*S. lycopersicum*	Genetic analysis	Butterbach *et al.* (2014)
rgs‐Cam	Regulator of gene silencing	CMV	2b	*N. tabacum*	Yeast two‐hybrid, transgenic overexpression	Anandalakshmi *et al.* (2000); Jeon *et al.* (2017)
PhOBF1	Transcription factor	TRV	NA	*Petunia hybrida*	VIGS	Sun *et al.* (2017)
Accumulation or activity of viral proteins						
NBR1	Autophagy cargo receptor	TuMV	HC‐Pro	*A. thaliana*	Genetic analysis	Hafren *et al.* (2018)
ATG7, ATG8	Autophagy	BSMV	*NA*	*N. benthamiana*	Yeast two‐hybrid, VIGS	Yang *et al.* (2018)
ATG8	Autophagy	CLCuMuV	ßC1	*N. benthamiana*	Yeast two‐hybrid, VIGS	Haxim *et al*. (2017)
rgs‐CaM	Immune receptor	CMV, TEV and TuMV	2b, HC‐Pro	*N. benthamiana*	Surface plasmon resonance	Jeon *et al.* (2017); Nakahara *et al.* (2012)
RNA replication						
GAPDH	Glycolysis	BaMV	3ʹ UTR	*N. benthamiana*	UV‐crosslinking to RdRp preparations	Prasanth *et al.* (2011)
Virion formation						
NBR1	Autophagy cargo receptor	CaMV	CP, virions	*A. thaliana*	Genetic analysis	Hafren *et al.* (2017)
PUS4	Pseudouridina synthase	BMV	Genomic RNA	*N. benthamiana*	Proteome array	Zhu *et al.* (2007)
Virus accumulation						
CYR1	NA	MYMIV	CP	*Vigna mungo*	Natural variation	Maiti *et al.* (2012)
NBR1	Autophagy cargo receptor	TuMV and WMV	HC‐Pro	*A. thaliana*	Genetic analysis	Hafren *et al.* (2018)
RFP1	Ubiquitination	TYLCCV	BC1	*N. tabacum*	Yeast two‐hybrid	Shen *et al.* (2016)
PSBP	Kinase	AMV	CP	*N. benthamiana*	Yeast two‐hybrid	Balasubramaniam *et al.* (2014)
Cell death						
N	Protein phosphatase	TMV	Helicase	*N. tabacum* 'Xanthi'	Transient expression	Abbink *et al.* (1998; Padgett *et al.* (1997)
RCY1	NA	CMV strain Y	CP	*A. thaliana*	Genetic mapping	Takahashi *et al.* (2001)
Rx1, Rx2	NA	PVX	CP	*S. tuberosum*	Transient expression	Bendahmane *et al.* (2000)
Tm‐2	NA	TMV	MP	*S. lycopersicum*	Genetic analysis	Meshi *et al.* (1989)
Tm‐2^2^	NA	ToMV	MP	*S. lycopersicum*	Cloning, transgenic expression, localization	Chen *et al.* (2017); Lanfermeijer *et al.* (2003)
RPP8	Protein binding	TCV	CP	*A. thaliana*	Cloning, transgenic expression	Cooley *et al.* (2000)
Rsv1	NA	SMV	P3 and HC‐Pro	*G. max*	Virus mutagenesis	Eggenberger *et al.* (2008)
Tsw	NA	TSWV	NSs	*Capsicum chinense*	Transient expression	de Ronde *et al.* (2013)
Sw5b	NA	TSWV	NSm	*S. tuberosum*	Transient and transgene expression	Mariana *et al.* (2014)

Virus names: alfalfa mosaic virus (AMV), bamboo mosaic virus (BaMV), barley stripe mosaic virus (BSMV), brome mosaic virus (BMV), cabbage leaf curl virus (CaLCuV), cauliflower mosaic virus (CaMV), cotton leaf curl multan virus (CLCuMuV), cymbidium ringspot virus (CymRSV), cucumber mosaic virus (CMV), cucumber necrosis virus (CNV), maize chlorotic mottle virus (MCMV), melon necrotic spot virus (MNSV), mungbean yellow mosaic india virus (MYMIV), oilseed rape mosaic virus (ORMV), pepper mild mottle virus (PMMoV), pepper mottle virus (PepMV), potato virus A (PVA), potato virus X (PVY), potato virus Y (PVY), rice stripe virus (RSV), soybean mosaic virus (SMV), sugarcane mosaic virus (SCMV), tobacco etch virus (TEV), tobacco mosaic virus (TMV), tobacco rattle virus (TRV), tomato bushy stunt virus (TBSV), tomato mosaic virus (ToMV), tomato ringspot virus (ToRSV), tomato yellow leaf curl virus (TYLCV), tomato spotted wilt virus (TSWV), tomato yellow leaf curl virus (ToYLCV), turnip crinkle virus (TCV), turnip mosaic virus (TuMV), turnip yellow mosaic virus (TYMV), watermelon mosaic virus (WMV).

Yeast: *Saccharomyces cerevisiae*.

## Viral Determinants of Infection

**Figure 2:**
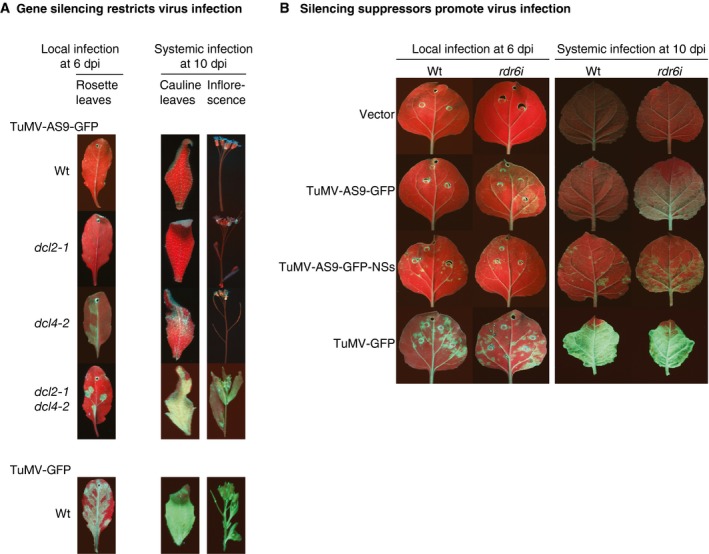
The balance between gene silencing and silencing suppression determines infection progression. *Arabidopsis thaliana* plants were mechanically inoculated with suppressor‐deficient turnip mosaic virus (TuMV)‐GFP or TuMV‐GFP. In *Nicotiana benthamiana* plants, infection was initiated by agroinfiltration. Pictures were taken under UV light. (A) In *A. thaliana*, Dicer‐like proteins 2 and 4 (DCL2 and DCL4) are core components of antiviral gene silencing and restrict virus infection in a tissue‐specific manner. In leaves, DCL4 is sufficient and DCL2 is dispensable. In the inflorescence, both DCL2 and DCL4 are necessary to restrict virus infection. TuMV‐encoded silencing suppressor (HC‐Pro) overcomes the antiviral effect of gene silencing and promotes the establishment of infection in leaves and the inflorescence. (B) In *N. bethamiana* RDR6 is an essential component of gene silencing. Suppressor‐deficient TuMV‐AS9‐GFP cannot infect wild‐type *N. benthamiana*. Local and systemic infection occurred by knocking down RDR6 in rdr6i plants, or by providing *in cis* the silencing suppressor from tomato spotted wilt virus. In normal and rd6i plants, local and systemic infection occur and the virus accumulates to high levels. Pathogenicity is determined by TuMV HC‐Pro.

RNA translation, genome replication, and virion formation and movement are core parts of the infection cycle of a plant by a virus (Ahlquist, 2006; Nelson and Citovsky, 2005). To accomplish these tasks, plant viruses encode replication, capsid, movement and auxiliary proteins. Additionally, to condition a cellular environment that is conducive to virus replication and movement, viral proteins target key components of antiviral immunity. Viral factors that determine the extent of infection and disease severity are considered pathogenicity determinants. Gene silencing suppressors are remarkable pathogenicity determinants of plant viruses. Virus‐encoded suppressors interfere with antiviral defence mechanisms mediated by gene silencing (Anandalakshmi *et al.*, 1998; Diaz‐Pendon *et al.*, 2007; Garcia‐Ruiz *et al.*, 2015; Jaubert *et al.*, 2011). Interestingly, the activity of several gene‐silencing suppressors promotes infection of heterologous viruses when expressed *in cis*‐, *trans*‐ or synergistic co‐infections (Garcia‐Ruiz *et al.*, 2018; Gupta *et al.*, 2018; Maliogka *et al.*, 2012) (Fig. 2).

## Host Determinants of Infection

Genome‐wide screens of *Saccharomyces cerevisiae* (yeast) replicating brome mosaic virus (BMV) (Kushner *et al.*, 2003) or tomato bushy stunt virus (TBSV) (Panavas *et al.*, 2005) showed that a compatible host contains both pro‐viral and antiviral factors that affect virus replication at the cellular level. Mutagenesis and genetic analyses in *Arabidopsis thaliana* (Arabidopsis) allowed identification of pro‐viral and antiviral factors that affect virus replication at the cellular level, cell‐to‐cell and systemic movement (Diaz‐Pendon *et al.*, 2007; Garcia‐Ruiz *et al.*, 2010; Guo *et al.*, 2017; Lellis *et al.*, 2002; Zhang *et al.*, 2019). Accordingly, viruses need pro‐viral host factors and are targeted by antiviral host factors. Pro‐viral host factors are necessary for essential steps of the infection cycle and work in synchrony with viral factors. In contrast, antiviral defence is mediated by host factors that target viral nucleic acids or proteins by multiple mechanisms such as autophagy, proteasome degradation, RNA decay and gene silencing (Fig. 1A).

## Compatibility and Susceptibility in Plant–Virus Interactions

The role of host and viral factors, and their availability in the cell during the establishment of infection, suggests a two‐step model to explain plant–virus interactions (Fig. 1B). Compatibility is determined by the availability of pro‐viral host factors. The absence of one or more pro‐viral factors results in incompatibility in a host plant (Lellis *et al.*, 2002). Viral proteins must be translated by the host translational machinery before replication can occur (Ahlquist, 2006; Machado *et al.*, 2017; Miller *et al.*, 2016). This feature makes viral RNA translation a critical determinant of the outcome in plant–virus interactions. An example is translation initiation factor eIF(iso)4E and potyviruses. Arabidopsis is susceptible to tobacco etch virus (TEV) and turnip mosaic virus (TuMV). However, mutant plants lacking eIF(iso)4E are immune to TEV and TuMV (Lellis *et al.*, 2002). Similarly, down‐regulation of eIF(iso)4E in plum confers resistance to plum pox virus (PPV) (Wang *et al.*, 2013). The mechanism is likely mediated by the lack of RNA translation to form potyviral polyproteins and possibly the lack of cell‐to‐cell movement (Contreras‐Paredes *et al.*, 2013; Lellis *et al.*, 2002). Similar effects have been detected for several plant species and their corresponding potyviruses (Sanfacon, 2015).

In compatible plant–virus combinations, susceptibility is determined by the balance between antiviral defence and suppression of antiviral defence (Fig. 1B). Defence responses may prevent infection from spreading to the entire plant, determining different levels of susceptibility to virus infection. If infection is stopped early, before the formation of local foci, the plant phenotype may be the same as that of a non‐host (Garcia‐Ruiz *et al.*, 2015; Lellis *et al.*, 2002; Qu *et al.*, 2008).

A genetic analysis of infection of Arabidopsis by green‐fluorescence protein‐tagged TuMV (TuMV‐GFP) illustrates a two‐step model in plant–virus interactions (Fig. 2A). Suppressor‐deficient (mutant helper component proteinase HC‐Pro) TuMV‐AS9‐GFP cannot infect wild‐type plants or *dcl2‐1* mutants. Infection is halted at the cellular level by gene silencing. However, suppressor‐deficient TuMV‐AS9‐GFP is able to infect *dcl4‐2* mutants, which lack the contribution of Dicer‐like protein 4 (DCL4) to gene silencing. Visible infection foci form and the virus moves systemically into cauline leaves without reaching the inflorescence. Interestingly, suppressor‐deficient TuMV‐AS9‐GFP is able to establish local and systemic infection of cauline leaves and inflorescence in *dcl2‐1 dcl4‐2* double mutants. Thus, in the absence of the HC‐Pro silencing suppression activity, gene silencing restricts infection in a tissue‐specific manner. In contrast, TuMV‐GFP establishes local and systemic infection, including the inflorescence, of Arabidopsis plants, wild‐type or mutants. Accordingly, the antiviral role of gene silencing is defeated by the TuMV‐encoded silencing suppressor HC‐Pro (Garcia‐Ruiz *et al.*, 2010).

A genetic analysis of infection of *Nicotiana benthamiana* by TuMV‐GFP further supports the two‐step model (Fig. 2B). Suppressor‐deficient TuMV‐AS9‐GFP cannot infect wild‐type *N. benthamiana*. In Arabidopsis and in *N. benthamiana*, RNA‐dependent RNA polymerase 6 (RDR6) is a core component of antiviral gene silencing (Garcia‐Ruiz *et al.*, 2010; Qu *et al.*, 2005, 2008; Yang *et al.*, 2004). In *N. benthamiana*, infection of the meristems by potato virus X (PVX) is prevented by RDR6 (Schwach *et al.*, 2005), and infection of the Arabidopsis meristems by TuMV is restricted by argonaute (AGO) proteins 1, AGO2 and AGO10 (Garcia‐Ruiz *et al.*, 2015).

Knockdown of RDR6 by RNA interference in *N. benthamiana* (rdr6i) (Schwach *et al.*, 2005) rescued local and systemic infection by TuMV‐AS9‐GFP. In an alternative approach, expression *in cis* of the NSs protein from tomato spotted wilt virus (TSWV) (Garcia‐Ruiz *et al.*, 2018) supported the establishment of local and systemic infection by TuMV‐AS9‐GFP (Fig. 2B). These observations show that tissue‐specific restriction of virus infection is determined by the balance between gene silencing and gene silencing suppression (Garcia‐Ruiz *et al.*, 2015, 2018; Schwach *et al.*, 2005).

## Host Genes with Antiviral Activity

Host factors with antiviral activity (Fig. 2) limit virus accumulation, movement or both, resulting in a virus‐resistant or tolerant phenotype that normally displays symptoms less severe than susceptible plants (Diaz‐Pendon *et al.*, 2007; Garcia‐Ruiz *et al.*, 2010; Huh *et al.*, 2013). For each part of the infection cycle, at least one host gene with antiviral activity has been identified (Table 1). Representative host factors are described below.

### Viral RNA translation

Translation of viral proteins from genomic RNA, subgenomic RNA or mRNA is dependent on cellular factors and the protein translation machinery. Being a critical step that determines the availability of viral proteins, both host and viral factors regulate translation (Ahlquist, 2006; Miller *et al.*, 2016; Sanfacon, 2015). In Arabidopsis, a leucine‐rich repeat receptor‐like kinase (NIK1) is a master regulator of translation. As a defence mechanism, using an NIK1‐dependent pathway, plants down‐regulate translation upon begomovirus infection. This effect results in a reduction in virus replication and accumulation. Remarkably, begomoviral nuclear shuttle protein (NSP) inactivates NIK1 to up‐regulate translation and promote susceptibility (Zorzatto *et al.*, 2015).

Using nucleotide sequences as recognition signatures, Arabidopsis Pumilio RNA binding protein 5 (APUM5) binds cucumber mosaic virus (CMV) and TuMV mRNA to inhibit translation. Accordingly, mutant plants lacking APUM5 accumulate CMV and TuMV to higher levels than plants harbouring wild‐type APUM5 (Huh *et al.*, 2013).

### Virus replication complex formation

After reaching the nucleus of infected cells, DNA viruses form minichromosomes that are replicated by cellular DNA‐dependent DNA polymerases (Ceniceros‐Ojeda *et al.*, 2016). In contrast, on cellular membranes, RNA viruses induce the formation of vesicles that contain RNA‐dependent RNA polymerases and genomic RNA, and are the sites of replication. Several cellular proteins that antagonize the formation of viral replication compartments have been identified and characterized (Table 1).

Phospholipids are crucial membrane components. Phosphatidic acid phosphohydrolase 1 (PAH1) limits phospholipid synthesis. Genetic analyses in yeast and *N. benthamiana* showed that PAH1 negatively regulates BMV and TBSV replication complex formation, resulting in reduced virus replication at the cellular level and reduced accumulation in plants (Chuang *et al.*, 2014; Zhang *et al.*, 2018).

### Accumulation or activity of replication proteins

Virus‐encoded RNA‐dependent RNA polymerases replicate the genome of RNA viruses and, if present, transcribe subgenomic RNAs that are essential for gene expression. Viral RNA‐dependent RNA polymerases contain a conserved GDD motif (Li *et al.*, 2018) and are targeted for degradation by autophagy protein 6 (ATG6 or Beclin1). Beclin1 is a core component of autophagy, interacting with and triggering degradation of the RNA‐dependent RNA polymerase (NIb) of several potyviruses, including TuMV, PPV, soybean mosaic virus (SMV) and TEV. Beclin1 also triggers degradation of the RNA‐dependent RNA polymerases of cucumber green mottle mosaic virus and pepino mosaic virus (Li *et al.*, 2018)*.* Additionally, in pepper (*Capsicum annum*), pathogenesis‐related protein 4c (Pvr4c) interacts with NIb and triggers cell death upon infection by pepper mottle virus or potato virus Y (PVY) (Kim *et al.*, 2015).

In tomato (*Solanum lycopersicum*), *tobacco mosaic virus resistance 1* (*Tm‐1*) confers resistance to tobacco mosaic virus (TMV) and to tomato mosaic virus (ToMV). *Tm‐1* encodes a protein that binds ToMV replication protein 103K and prevents its normal activity (Ishibashi and Ishikawa, 2013; Ishibashi *et al.*, 2007).

### RNA replication

Glycolytic enzyme glyceraldehyde 3‐phosphate dehydrogenase (GAPDH) binds the 3′ UTR of bamboo mosaic virus (BaMV) and satellite BaMV RNAs. This interaction down‐regulates BaMV negative‐strand RNA synthesis. Accordingly, GAPDH knockdown in *N. benthamiana* enhanced accumulation of BaMV. In contrast, GAPDH overexpression reduced BaMV accumulation (Prasanth *et al.*, 2011). GAPDH has the opposite effect on TBSV replication. GAPDH preferentially binds to the 3′ end of negative‐strand TBSV RNA, retaining it in the replication complex to promote positive‐strand RNA synthesis. This activity results in asymmetric RNA replication characterized by higher synthesis and accumulation of positive‐ over negative‐strand genomic RNA, which is normal in TBSV replication (Wang and Nagy, 2008)

### mRNA stability

Viruses express their genes through mRNA (Ahlquist, 2006). After translation, cellular and viral mRNAs are deadenylated, decapped and cleaved 5′ to 3′ by exoribonuclease 4 (XRN4) through the decapping‐dependent RNA decay pathway (Thran *et al.*, 2012; Tsuzuki *et al.*, 2017). Recent genetic analyses showed that RNA decay has an antiviral role that limits virus accumulation and may contribute to plant recovery from virus‐induced symptoms (Li and Wang, 2018; Ma *et al.*, 2015; Moon and Wilusz, 2013; Tsuzuki *et al.*, 2017). RNA decay and RNA silencing seem to act in coordination to suppress virus infection, and their activities partially overlap (Li and Wang, 2018; Peng *et al.*, 2011).

Consistent with the antiviral role of RNA decay, potyviral HC‐Pro and genome‐linked protein (VPg) are silencing suppressor proteins that interfere with both gene silencing and mRNA decay. Interference with mRNA decay occurs through interactions with XRN4 and decapping protein 2 (DCP2), respectively, two core components of the 5′ to 3′ RNA decay pathway (Li and Wang, 2018).

### Virus movement

Plant viruses move cell to cell as virions or nucleoprotein complexes through plasmodesmata. As a critical component of this process, plant viruses encode movement proteins that increase the plasmodesmata size exclusion limit and/or form microtubules (Taliansky *et al*., 2008). Several host factors that antagonize virus movement have been identified. They target viral proteins and RNA, or trigger cell death (Table 1).

Plants encode mechanosensitive ion channels that regulate ion movement across cells. In Arabidopsis, ESC1 encodes a piezo protein that functions as a mechanosensitive Ca^2+^ permeable channel and limits systemic infection of CMV and TuMV (Zhang *et al.*, 2019).

Systemic movement of TEV, and some isolates of PPV and lettuce mosaic virus (Decroocq *et al.*, 2009), is restricted by restricted‐TEV‐movement (RTM) genes *RTM1*, *RTM2* and *RTM3*. These genes are expressed in phloem sieve elements and interact with the viral coat protein (Chisholm *et al.*, 2001). Interestingly, resistance‐breaking isolates had mutations in the N‐terminus of the coat protein (Decroocq *et al.*, 2009).

BTR1 is a ribonucleoprotein K‐homology RNA‐binding protein that binds ToMV genomic RNA and restricts cell‐to‐cell movement (Fujisaki and Ishikawa, 2008). In potato (*S. tuberosum*) plants the *Ny‐1* gene confers resistance to PVY by triggering cell death at the infection sites, limiting cell‐to‐cell movement (Lukan *et al.*, 2018).

### Gene silencing

In plants, gene silencing is an essential mechanism of antiviral defence. Gene silencing targets viral RNA for degradation or translational repression. The result is restriction of virus replication and movement, and recovery from virus‐induced symptoms (Korner *et al.*, 2018; Szittya and Burgyan, 2013). Gene silencing targets DNA and RNA viruses, satellite RNA viruses and viroids (Blevins *et al.*, 2006; Diaz‐Pendon *et al.*, 2007; Minoia *et al.*, 2014; Shimura *et al.*, 2011). All viruses express their genes and/or replicate their genome through an RNA intermediate (Ahlquist, 2006). This feature exposes viruses to gene silencing.

The core components of gene silencing include Dicer‐Like (DCL), Argonaute (AGO), double‐stranded RNA binding (DRB) and RNA‐dependent‐RNA‐polymerase (RDR) proteins. These proteins are conserved across plants (Incarbone and Dunoyer, 2013; Szittya and Burgyan, 2013; Zvereva and Pooggin, 2012). A signature feature of antiviral gene silencing is the accumulation of virus‐derived small interfering RNAs (siRNAs) in infected plants. Viral RNA is processed by DCL proteins into siRNAs that are 21 to 24 nucleotides long. Virus‐derived siRNAs are loaded into AGO proteins, programming them for specific slicing or translational repression of viral RNA (Garcia‐Ruiz *et al.*, 2015; Karran and Sanfacon, 2014; Schuck *et al.*, 2013). Accordingly, viral RNA is targeted by both DCL and AGO proteins.

Antiviral gene silencing might be triggered by viral RNA replication intermediates, self‐complementary sequences forming hairpin structures in viral single‐stranded RNA, and by products of overlapping transcription (Pantaleo *et al.*, 2007; Szittya and Burgyan, 2013). DCL proteins process double‐stranded virus RNA into primary virus‐derived siRNAs that are necessary but not sufficient to prevent virus infection. Establishment of an antiviral state requires silencing amplification by plant RNA‐dependent RNA polymerases that synthesize double‐stranded RNA from single‐stranded viral RNA (Diaz‐Pendon *et al.*, 2007; Garcia‐Ruiz *et al.*, 2010).

Virus‐derived siRNA profiling has demonstrated that, in compatible plant–virus interactions, the entire genome of positive‐strand and negative‐strand RNA viruses is targeted by gene silencing in both monocot and dicot plants (Donaire *et al.*, 2009; Garcia‐Ruiz *et al.*, 2015; Margaria *et al.*, 2015; Tatineni *et al.*, 2014; Wang *et al.*, 2011; Xia *et al.*, 2016). However, gene silencing is not enough to restrict virus infection. That is due to the inhibitory activity of virus‐encoded gene silencing suppressors. Suppressors condition susceptibility, promote virus replication and movement, and promote symptom development by interfering with endogenous and antiviral gene silencing (Burgyan and Havelda, 2011; Garcia‐Ruiz *et al.*, 2018; Kasschau *et al.*, 2003). The mechanisms of silencing suppression include triggering the degradation of core components of gene silencing such as DCL, AGO, RDR6 and suppressor of gene silencing 3 (SGS3) proteins, and binding of both virus‐derived and cellular siRNAs including micro‐RNAs (miRNAs) (Burgyan and Havelda, 2011; Garcia‐Ruiz *et al.*, 2015; Del Toro *et al.*, 2017). These effects prevent the biogenesis and/or activity of virus‐derived and cellular siRNAs. Plant development and response to abiotic and biotic stress is in part regulated by miRNAs and other siRNAs. Accordingly, virus‐encoded gene silencing suppressors are determinants of symptom development (Garcia‐Ruiz *et al.*, 2018; Kasschau *et al.*, 2003). Furthermore, viral silencing suppressors impact virus accumulation and the spatial distribution of virus infection (Garcia‐Ruiz *et al.*, 2018), and are frequently determinants of host range (Garcia‐Ruiz *et al.*, 2015; Jaubert *et al.*, 2011; Li *et al.*, 2014).

The antiviral role of gene silencing was unambiguously demonstrated using viruses lacking gene silencing suppressors (Diaz‐Pendon *et al.*, 2007; Garcia‐Ruiz *et al.*, 2010, 2015; Pantaleo *et al.*, 2007; Qu *et al.*, 2008). Turnip crinkle virus (TCV), TBSV, CMV and TuMV accumulate to similar levels in wild‐type plants and in mutant plants lacking core components of the silencing machinery. However, suppressor‐deficient viruses cannot infect wild‐type plants. Instead, suppressor‐deficient viruses can only infect plants lacking core gene silencing components (Fig. 2). These genetic systems have been used to identify and characterize components of gene silencing (Diaz‐Pendon *et al.*, 2007; Garcia‐Ruiz *et al.*, 2010, 2015; Pantaleo *et al.*, 2007; Qu *et al.*, 2008).

As illustrated by genetic analyses using suppressor‐deficient TuMV‐AS9‐GFP, antiviral gene silencing restricts virus infection and movement in a tissue‐specific manner. In Arabidopsis plants lacking DCL4, AGO2, RDR1 or RDR6, TuMV‐AS9‐GFP established local infection and moved systemically into non‐inoculated leaves, without reaching the inflorescence. Systemic infection of the inflorescence only occurred in the absence of both DCL2 and DCL4, or RDR1 and RDR6, or AGO1, AGO2 and AGO10 (Garcia‐Ruiz *et al.*, 2010, 2015).

To prevent their inhibitory effect on gene silencing, several plant factors target virus‐encoded silencing suppressors or regulate expression of gene silencing components (Table 1), as illustrated by the following examples. In *Nicotiana tabacum*, a calmodulin‐like protein (rgs‐CaM) binds to and, via autophagy directs degradation of, 2b, the silencing suppressor in CMV (Jeon *et al.*, 2017). Autophagy cargo receptor NBR1 targets potyviral HC‐Pro for degradation, thus affecting silencing suppression and reducing accumulation of TuMV and watermelon mosaic virus (WMV). Interestingly, TuMV VPg and 6K2 prevent NBR1‐dependent degradation of HC‐Pro (Hafren *et al.*, 2018).

In petunia (*Petunia hybrida*), PhOBF1, a leucine transcription factor, is up‐regulated by tobacco rattle virus (TRV) infection. PhOBF1 is a positive regulator of salicylic acid biosynthesis and of core components of gene silencing: DCL, AGO and RDRs. Thus, PhOBF1 enhances antiviral responses to TRV (Sun *et al.*, 2017). In tomato, the *Ty‐1* gene encodes an RNA‐dependent RNA polymerase that confers resistance to geminiviruses by enhancing transcriptional gene silencing (Butterbach *et al.*, 2014).

### Virion assembly and disassembly

In BMV, the negative‐strand RNA core promoter consists of a short stem with a three‐nucleotide loop that forms a clamp adenine motif. An array of 5000 yeast proteins was screened for proteins that bind the clamp adenine motif. Pseudouridine synthase 4 (PUS4) was identified. Functional characterization in *N. benthamiana* showed that PUS4 binding to BMV positive‐strand RNA prevented encapsidation, resulting in a slight reduction in viral RNA accumulation and a drastic reduction in BMV systemic movement (Zhu *et al.*, 2007).

### Host factors that condition virus resistance by undetermined mechanisms

For a growing number of plant–virus combinations, reduced virus accumulation has been observed in the presence of genes with antiviral activity although the part of the infection cycle that is affected has not been identified (Table 1). In these cases, virus infection triggers a hypersensitive response that results in the formation of necrotic lesions. Cell death might reduce virus movement and confine the virus to the infection sites and surrounding cells, but is not sufficient to prevent virus movement out of the cell death zone (Lukan *et al.*, 2018).

The following plant–virus combinations are examples of host factors that condition virus resistance by undetermined mechanisms. The *N* resistance gene from *Nicotiana glutinosa* was introduced into *N. tabacum* and confers resistance to TMV (Levy *et al.*, 2004). The N resistance protein is a receptor that contains three essential domains: a Toll‐interleukin‐1 (TIR), a nucleotide‐binding site (NBS) and a leucine‐rich repeat (LRR) (Dinesh‐Kumar *et al.*, 2000). Transcription and alternative splicing of the *N* gene is stimulated by TMV infection (Levy *et al.*, 2004), the protein coded by the *N* gene recognizes the helicase domain in TMV replication protein 126‐kD, and triggers a hypersensitive response visible as local necrotic lesions. As a result, TMV infection is restricted to cells surrounding the entry site (Abbink *et al.*, 1998; Levy *et al.*, 2004; Padgett *et al.*, 1997).

The arginine‐rich cyclin 1 (*RCY1*) gene in Arabidopsis recognizes the coat protein in CMV strain Y and triggers local cell death (Takahashi *et al.*, 2001). Similarly, TSWV infection triggers cell death in plants carrying the *Tsw* and *Sw5b* genes, which recognize the NSs or NSm proteins, respectively (Mariana *et al.*, 2014). Likewise, the *Tm‐2* ^*2*^ gene in tomato encodes a leucine‐rich protein that interacts with the movement protein and confers resistance to tobamoviruses, including TMV. The response is mediated by a hypersensitive response and localized cell death (Chen *et al.*, 2017). In soybean (*Glycine max*), the *Rsv1* gene confers resistance to SMV strain N. SMV strain G7 is not affected. Both P3 and HC‐Pro mediate *Rsv1*‐dependent restriction of SMV strain N (Eggenberger *et al.*, 2008). A component of the oxygen‐evolving complex pathosystem II, PSBP, interacts with alfalfa mosaic virus coat protein and delays activation of antiviral responses mediated by reactive oxygen species (Balasubramaniam *et al.*, 2014).

## Identification of Host Factors with Antiviral Activity

Host factors with antiviral activity have been identified and characterized using several experimental approaches (Table 1). For a small number of cases, natural genetic variation in plant populations has been used to identify, map and clone genes with antiviral activity (Lukan *et al.*, 2018; Maiti *et al.*, 2012; Szajko *et al.*, 2008). However, experimental model systems based on yeast, Arabidopsis and *N. benthamiana* have contributed most of the genes with antiviral activity known to date (Table 1). These experimental systems allow systematic genetic analysis of virus–host interactions. Using yeast, genome‐wide screens have been conducted for BMV and TBSV. Based on the Arabidopsis mutant collection, multiple screens have been done for gene families such as DCL, AGO, RDRs, RNA decay or autophagy mutants (Blevins *et al.*, 2006; Diaz‐Pendon *et al.*, 2007; Garcia‐Ruiz *et al.*, 2015; Jaubert *et al.*, 2011; Qu *et al.*, 2008).

Based on the concept that host and viral factors colocalize and may interact, yeast‐two hybrid assays and cell fractionation or immunoprecipitation followed by mass spectrometry has led to the identification of several genes with antiviral activity (Fujisaki and Ishikawa, 2008; Ishibashi *et al.*, 2007). These assays involved a virus natural host or an experimental host (Table 1).

Functional characterization of the genes identified has been done using loss‐of‐function or gain‐of‐function mutants in Arabidopsis, *N. benthamiana* or natural hosts. Additionally, virus‐induced gene silencing (VIGS) has been widely used to down‐regulate genes in *N. benthamiana*. In both Arabidopsis and *N. benthamiana*, transient or transgenic expression has been used to validate the antiviral activity of a growing number of genes (Haxim *et al*., 2017; Jaag and Nagy, 2009; Sun *et al.*, 2017).

## Factors Essential and Non‐Essential for Host Survival

Genes with antiviral activity might be essential or non‐essential for plant survival. Non‐essential genes affect virus replication or movement without affecting the host (Table 1). However, essential genes cannot be removed from the host. AGO1 participates in antiviral defence (Garcia‐Ruiz *et al.*, 2015; Qu *et al.*, 2008; Wang *et al.*, 2011) and is essential for miRNA‐dependent regulation of gene expression and development. Accordingly, ago1 null mutants show severe developmental phonotypes and are sterile. Hypomorphic *ago1* mutant alleles retain part of their activity and have been used to genetically characterize the role of AGO1 in antiviral defence (Morel *et al.*, 2002). In contrast, DCL2 and DCL4 are non‐essential, are redundant to each other, and single and double mutants show only mild leaf malformation (Diaz‐Pendon *et al.*, 2007; Garcia‐Ruiz *et al.*, 2010).

Conditional repression of expression or temperature‐sensitive expression were used to determine the role of yeast essential genes in BMV and TBSV replication (Gancarz *et al.*, 2011; Nawaz‐ul‐Rehman *et al.*, 2013). These genetic analyses identified 19 essential yeast genes that antagonized BMV (Gancarz *et al.*, 2011) or TBSV replication (Nawaz‐ul‐Rehman *et al.*, 2013).

## Conclusions

The infection cycle of a plant by a virus is genetically determined by viral factors, cellular factors and their interaction. Viruses use cellular factors and resources to replicate and move. Viral protein or nucleic acids are targeted by antiviral immunity (Fig. 1A). A two‐step model for plant–virus interactions explains plant susceptibility to viruses (Fig. 1B). Initially, establishment of infection is determined by the level of plant–virus compatibility. Incompatibility might result from the lack of pro‐viral factors, while compatibility is determined by the availability of pro‐viral host factors. Subsequently, in compatible plant–virus combinations susceptibility is determined by the balance between antiviral defence and suppression of antiviral defence (Fig. 1B). Strong antiviral defence may stop infection at any point before spreading to the entire plant. This range results in plants with different levels of susceptibility. The lowest level of susceptibility, resulting from arrest of infection at the initially infected cell, is difficult to distinguish from an incompatible interaction that occurs in a non‐host (Fig. 1B). Susceptible hosts harbour both pro‐viral factors and factors with antiviral activity (Table 1). Their functional characterization has improved our understanding of the mechanisms of virus pathogenicity and antiviral defence in plant–virus interactions.

## Future Directions

Host genes with antiviral activity provide an interesting option to develop genetic resistance to viruses in crops. However, viruses have a remarkable ability to mutate and are rapidly evolving (Duffy, 2018). Virus‐resistant plants select for variants capable of breaking genetic resistance. An example is the emergence of tomato brown rugose fruit virus (ToBRV), described in 2016. ToBRV originated from a recombination event between TMV and tomato mild mottle virus (ToMMV) (Salem *et al.*, 2016). Interestingly, within a year, a ToBRV isolate that broke the *Tm‐2* ^*2*^‐dependent resistance was identified (Luria *et al.*, 2017).

A complementary or alternative approach to the deployment of genes with antiviral activity is the identification, characterization and deployment of pro‐viral factors that determine susceptibility to plant viruses (Garcia‐Ruiz, 2018).

To date, genome‐wide screens and genetic analysis have been done mainly in model viruses using heterologous hosts and/or model plant systems (Table 1). Current advances in genome editing (Zhe *et al.*, 2018) make it possible to implement genetic analysis in crop plants. Genome editing in combination with epitope‐tagging of viral proteins, either individually or in the context of an infectious clone, make it currently possible to identify and characterize host genes with antiviral activity and pro‐viral genes crop plants. Thus, it is safe to predict that viruses causing devastating diseases in staple crops will receive more attention in the near future. This includes orthotospoviruses, potyviruses, tobamoviruses and geminiviruses. Prime examples are the causal agents of maize lethal necrosis, maize chlorotic mottle virus and sugarcane mosaic virus (Wamaitha *et al.*, 2018), and recently described ToBRV (Luria *et al.*, 2017; Salem *et al.*, 2016).

## Conflicts of Interest

The author declares no conflict of interest. The founding sponsors had no role in the design of the study, in the collection, analyses or interpretation of data, in the writing of the manuscript or in the decision to publish the results.

## Author Contributions

Hernan Garcia‐Ruiz conceived and wrote the paper.
